# Convergence Analysis of Particle Swarm Optimizer and Its Improved Algorithm Based on Velocity Differential Evolution

**DOI:** 10.1155/2013/384125

**Published:** 2013-08-28

**Authors:** Hongtao Ye, Wenguang Luo, Zhenqiang Li

**Affiliations:** School of Electrical and Information Engineering, Guangxi University of Science and Technology, Liuzhou 545006, China

## Abstract

This paper presents an analysis of the relationship of particle velocity and convergence of the particle swarm optimization. Its premature convergence is due to the decrease of particle velocity in search space that leads to a total implosion and ultimately fitness stagnation of the swarm. An improved algorithm which introduces a velocity differential evolution (DE) strategy for the hierarchical particle swarm optimization (H-PSO) is proposed to improve its performance. The DE is employed to regulate the particle velocity rather than the traditional particle position in case that the optimal result has not improved after several iterations. The benchmark functions will be illustrated to demonstrate the effectiveness of the proposed method.

## 1. Introduction

Algorithms to tackle optimization problems include not only classical techniques such as dynamic programming, branch-and-bound, and gradient-based methods, but also more recent techniques such as metaheuristics [1]. Among the existing metaheuristic algorithms, the particle swarm optimization (PSO) algorithm is a population-based optimization technique developed by Kennedy and Eberhart in 1995 [2]. The PSO has resulted in a large number of variants of the standard PSO. Some variants are designed to deal with specific applications [3–6], and others are generalized for numerical optimization [7–10]. A hierarchical version of PSO (H-PSO) has been proposed by Janson and Middendorf [10]. In H-PSO, all particles are arranged in a tree that forms the hierarchy. A particle is influenced by its own best position and the best position particle in its neighborhood. It was shown that H-PSO performed very well compared to the standard PSO on unimodal and multimodal test functions [10, 11]. H-PSO presents the advantage of being conceptually very simple and requiring low computation time. However, the main disadvantage of H-PSO is the risk of a premature search convergence, especially in complex multiple peak search problems.

A number of algorithms combined various algorithmic components, often originating from algorithms of other research areas on optimization. These approaches are commonly referred to as hybrid meta-heuristics [12]. The surveys on hybrid algorithms that combine the PSO and differential evolution (DE) [13] were presented recently [14, 15]. These PSO-DE hybrids usually employ DE to adjust the particle position. But the convergence performance is dependent on the particle velocity. Limiting the velocity can help the particle to get out of local optima traps [16, 17]. In this paper, we will combine these two optimization algorithms and propose the novel hybrid algorithm H-PSO-DE. The DE is employed to regulate the particle velocity rather than the traditional particle position in case that the optimal result has not improved after several iterations. The hybrid algorithm aims to aggregate the advantages of both algorithms to efficiently tackle the optimization problem.

The remainder of this paper is organized as follows.  Section 2 briefly describes the basic operations of the PSO, H-PSO, and DE algorithms.  Section 3 presents an analysis of the relationship of particle velocity and convergence.  Section 4 provides the hybrid optimization method: H-PSO-DE.  Section 5 reveals the simulations and analysis of H-PSO-DE in solving unconstrained optimization problems. Finally, conclusions are given in Section 6.

## 2. The PSO, H-PSO, and DE Algorithms

### 2.1. The PSO Algorithm

The PSO [18–20] is a stochastic population-based optimization approach. Each particle is a *D*-dimensional vector, and it consists of a position vector *x*_*n*_, which represents a candidate solution of the optimization problem, a velocity vector *v*_*n*_, and a memory vector *y*_*n*_, which is the best candidate solution encountered by the particle. The velocity and position of the particle are updated in every dimension *d*  (1 ⩽ *d* ⩽ *D*) by

(1) vn,d(t+1)=wvn,d(t)+c1r1(yn,d(t)−xn,d(t))+c2r2(yp(n),d(t)−xn,d(t)),

(2) $$\begin{array}{c} x_{n,d}\left(t+1\right)\;=x_{n,d}\left(t\right)+v_{n,d}\left(t+1\right), \end{array}$$

where *w* is the inertia weight, which determines how much of the previous velocity the particle is preserved. *c*_1_ and *c*_2_ are positive constants. *r*_1_ and *r*_2_ are randomly chosen numbers uniformly distributed in the interval [0, 1]. *y*_*p*(*n*)_ represents the best position achieved by any member of the population.

### 2.2. The H-PSO Algorithm

In H-PSO [21], all particles are arranged in a hierarchy. The hierarchy is defined by the *height h*, the* branching degree bd*, and the *total number of nodes tnn* of the corresponding tree.

In H-PSO, the iteration starts with the evaluation of the objective function of each particle at its current position. Then, the new velocity vectors and the new positions for the particles are determined. This means that for particle *n*, the value of *y*_*p*(*n*)_ in (1) equals *y*_*m*_, with *m* being the particle in the parent node of the node of particle *n*. H-PSO uses *y*_*p*(*n*)_ = *y*_*n*_ only when particle *n* is in the root. If the function value of a particle *n* is better than the function value at its personal best position so far, then the new position is stored in *y*_*n*_. For each particle *n* in a node of the tree, its own best solution is compared to the best solution found by the particles in the child nodes *S*(*n*). If the best of these particles *m* is better than particle *n*, then particles *n* and *m* swap their places within the hierarchy.

### 2.3. The DE Algorithm

The DE [11, 13, 22] is a stochastic parallel direct search method. More specifically, DE's basic strategy can be summarized as follows.

*Initialization*. DE begins with a randomly initiated population of *N* *D*-dimensional parameter vectors *x*_*i*,*g*_, *i* = 1,2,…, *N* as a population for each generation *g*. The initial population (*g* = 0) of the *j*th parameter of the *i*th vector is

(3) $$\begin{array}{c} x_{j,i,0}=x_{j,\min}+{\text{rand}}_{i,j}\left[0,1\right]\cdot \left(x_{j,\max}-x_{j,\min}\right), \end{array}$$

where *x*_*j*,min⁡_ and *x*_*j*,max⁡_ indicate the lower and upper bounds, respectively. rand_*i*,*j*_[0,1] is a uniformly distributed random number lying between 0 and 1.

*Mutation*. DE mutates and recombines the population to produce a population of *N* trial vectors. Specifically, for each individual *x*_*i*,*g*_, a mutant vector *υ*_*i*,*g*_ is generated according to

(4) $$\begin{array}{c} \upsilon_{i,g}=x_{r_{1}^{i},g}+F\cdot \left(x_{r_{2}^{i},g}-x_{r_{3}^{i},g}\right), \end{array}$$

where *F*, commonly known as scale factor, is a positive real number. Three other random individuals *x*_*r*_1_^*i*^,*g*_, *x*_*r*_2_^*i*^,*g*_, and *x*_*r*_3_^*i*^,*g*_ are sampled randomly from the current population such that *r*_1_^*i*^, *r*_2_^*i*^, *r*_3_^*i*^ ∈ {1,2,…, *N*}, and *i* ≠ *r*_1_^*i*^ ≠ *r*_2_^*i*^ ≠ *r*_3_^*i*^.

*Crossover*. DE crosses each vector with a mutant vector:

(5) $$\begin{array}{c} u_{j,i,g}=\{\begin{array}{cc} \upsilon_{j,i,g}, & \text{if}\;\;\left({\text{rand}}_{i,j}\left[0,1\right]\leq C_{r}\;\;\text{or}\;\;j=j_{\text{rand}}\right),\\ x_{j,i,g}, & \text{otherwise,} \end{array} \end{array}$$

where *C*_*r*_ is called the crossover rate.

*Selection*. To decide whether or not it should become a member of generation *g* + 1, the trial vector *υ*_*i*,*g*_ is compared to the target vector *x*_*i*,*g*_ using the greedy criterion. The selection operation is described as

(6) $$\begin{array}{c} x_{i,g+1}=\{\begin{array}{cc} u_{i,g}, & \text{if}\;\;f\left(u_{i,g}\right)\leq f\left(x_{i,g}\right),\\ x_{i,g}, & \text{otherwise,} \end{array} \end{array}$$

where *f*(*x*) is the objective function to be minimized.

## 3. Relationship of Particle Velocity and Convergence

This section presents an analysis of the relationship of particle velocity and convergence. 

Substituting (1) into (2) results in

(7) xn,d(t+1)=xn,d(t)+wvn,d(t)+c1r1(yn,d(t)−xn,d(t)) +c2r2(yp(n),d(t)−xn,d(t)).

From (2), it is known that

(8) $$\begin{array}{c} v_{n,d}\left(t\right)=x_{n,d}\left(t\right)-x_{n,d}\left(t-1\right). \end{array}$$

Substituting (8) into (7) results in

(9) xn,d(t+1)=(1+w−c1r1−c2r2)xn,d(t)−wxn,d(t−1) +c1r1yn,d(t)+c2r2yp(n),d(t).

This recurrence relation can be written as a matrix-vector product, so that

(10) [xn,d(t+1)xn,d(t)1] =[1+w−c1r1−c2r2−wc1r1yn,d(t)+c2r2yp(n),d(t)100001]  ×[xn,d(t)xn,d(t−1)1].

The characteristic polynomial of the matrix in (10) is (1 − *λ*)(*w* − *λ*(1 + *w* − *c*_1_ *r*_1_ − *c*_2_ *r*_2_) + *λ* ^2^), which has a trivial root of *λ* = 1 and two other solutions

(11) $$\begin{array}{c} \alpha =\frac{\left(1+w-c_{1}r_{1}-c_{2}r_{2}+\gamma \right)}{2},\\ \beta =\frac{\left(1+w-c_{1}r_{1}-c_{2}r_{2}-\gamma \right)}{2}, \end{array}$$

where $\gamma =\sqrt{{{(1+w-c}_{1}r_{1}-c_{2}r_{2})}^{2}-4w}$.

Note that *α* and *β* are both eigenvalues of the matrix in (10). The explicit form of the recurrence relation (9) is then given by

(12) $$\begin{array}{c} x_{n,d}\left(t\right)=k_{1}+k_{2}\alpha^{t}+k_{3}\beta^{t}, \end{array}$$

where *k*_1_, *k*_2_, and *k*_3_ are constants determined by the initial conditions of the system.

Substituting (12) into (8) results in

(13) $$\begin{array}{c} v_{n,d}\left(t\right)=h_{1}\alpha^{t}+h_{2}\beta^{t}, \end{array}$$

where *h*_1_ = *k*_2_(1 − 1/*α*), *h*_2_ = *k*_3_(1 − 1/*β*).

Consider

(14) $$\begin{array}{c} \underset{t\to \infty }{\lim}v_{n,d}\left(t\right)=\underset{t\to \infty }{\lim}\left(h_{1}\alpha^{t}+h_{2}\beta^{t}\right), \end{array}$$

(15) lim⁡t→∞vn,d(t) ={0,if  max(||α||,||β||)<1,h1or  h2  or  h1+h2,if  max(||α||,||β||)=1.

Equation (15) implies that if the PSO algorithm is convergent, the velocity of the particles will decrease to zero or stay unchanged until the end of the iteration.

## 4. The Proposed H-PSO-DE Algorithm

The main idea of the hybrid H-PSO-DE algorithm is to employ the DE to regulate the particle velocity rather than the traditional particle position in case that the optimal result has not improved after several iterations. If the swarm is going to be in equilibrium, the evolution process will be stagnated as time goes on. To prevent the trend, if the stagnating step of evolution process *g*_0_ is larger than threshold value *G*_0_, the particle velocity performs mutation operators. The velocity and position of the particles are updated as follows.

If (rand() < *C*_*r*_ or *d* = = *k*, *k* ∈ [1, *D*]), then

(16) $$\begin{array}{c} v_{n,d}^{\prime }=v_{n,d}+F\cdot \left(v_{1,d}-v_{2,d}\right),\\ x_{n,d}^{\prime }=x_{n,d}+v_{n,d}^{\prime }, \end{array}$$

where *x*_*n*,*d*_ = *r* · *y*_*n*,*d*_ + (1 − *r*) · *y*_*p*(*n*),*d*_, *r* is a random number in the interval [0, 1], and *v*_1,*d*_ and *v*_2,*d*_ are sampled randomly from *v*_*n*_. 

The procedure for H-PSO-DE algorithm is presented in Algorithm 1. 

## 5. Simulations and Results

**Figure 1:**
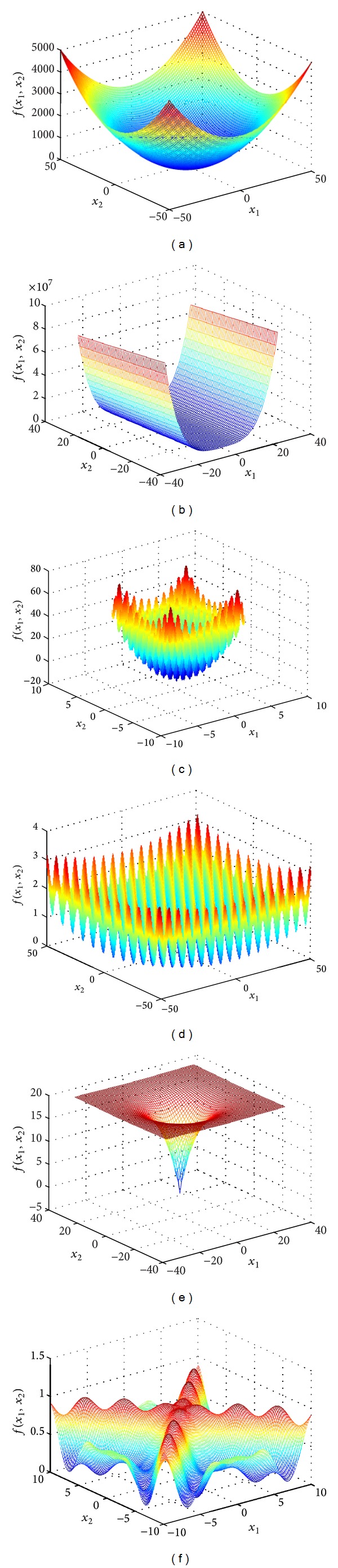
An illustration for 2-dimensional landscapes of the test functions. (a) Sphere function; (b) Rosenbrock function; (c) Rastrigin function; (d) Griewank function; (e) Ackley function; and (f) Schaffer's F6.

In this section, we present a simulation study to validate the proposed H-PSO-DE algorithm. A set of test functions that are commonly used in the field of continuous function optimization is listed in the appendix. They are a set of curvilinear functions for difficult unconstrained minimization problems. For illustration, the landscapes of two-dimensional versions of the six functions are depicted in Figure 1. The first two functions (Sphere and Rosenbrock) are unimodal functions, and they have a single local optimum that is also the global optimum. The remaining functions are multimodal, and they have several local optima. Note that the dimensional increase of these scalable functions does not change their basic features.

**Figure 2:**
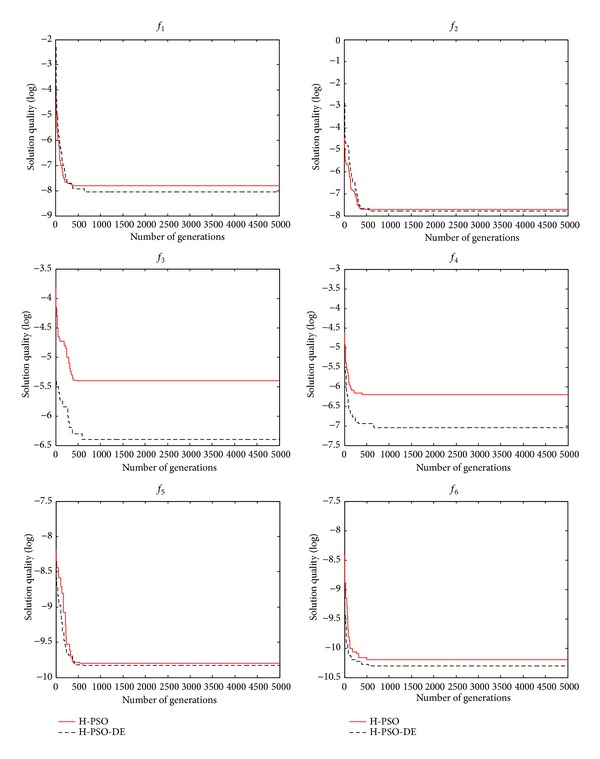
Convergence graph of the H-PSO and the H-PSO-DE for *f*_1_–*f*_6_.

In our experiments, the H-PSO uses the parameter values *w* = 0.729, and *c*_1_ = *c*_2_ = 1.494 as suggested in [23] for a faster convergence rate. The population size that has been used is *N* = 21. The maximal number of generations uses *G* = 5000. The remainder parameters are set as *C*_*r*_ = 0.5, *F* = 0.6, *r* = 0.5, *G*_0_ = 8, *h* = 3, and *bd* = 4. Thirty independent runs were carried out. The convergence behavior of the H-PSO is shown in Figure 2. For comparison purpose, the H-PSO-DE is also given in the same figure. As shown in Figure 2, the convergence performance of the H-PSO-DE is better than the H-PSO. H-PSO-DE is compared with H-PSO, DE, and PSO-DE [1] in terms of the selected performance metrics, such as the mean, maximum, and minimum values. In DE, we use DE/rand/1/bin strategy (*C*_*r*_ = 0.5, *F* = 0.6). As shown in Tables 1, 2, and 3, the H-PSO-DE outperforms H-PSO, DE, and PSO-DE. The H-PSO-DE is quite competitive when compared with the other existing methods.

## 6. Conclusions

In this paper, a new method named H-PSO-DE is proposed to solve optimization problems, which improves the performance of the H-PSO by incorporating DE. In H-PSO-DE, when the evolution process is stagnated for several generations, all the particles may lose the ability of finding a better solution. Then, the DE is employed to regulate the particle velocity to avoid wasting too much calculation time for vain search, so the searching efficiency of the H-PSO-DE is improved greatly. The H-PSO-DE is compared on test functions with H-PSO, DE, and PSO-DE. It is shown that H-PSO-DE performs significantly better.

## Figures and Tables

**Algorithm 1:**
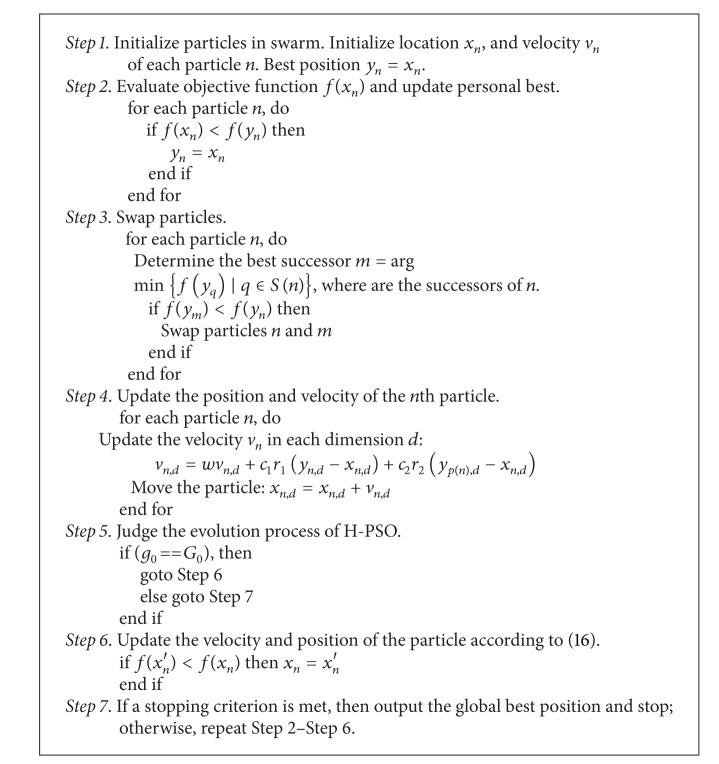
Procedure for the H-PSO-DE.

**Table 1:** Comparing the mean value of H-PSO-DE with respect to the other state-of-the-art algorithms.

Function	Mean value of the solution			
	H-PSO-DE	H-PSO	DE	PSO-DE
*f*_1_	2.06*e* − 8	6.13*e* − 7	7.29*e* − 5	6.80*e* − 7
*f*_2_	3.23*e* − 7	5.14*e* − 7	2.14*e* − 6	7.95*e* − 7
*f*_3_	1.77*e* − 6	3.26*e* − 5	5.21*e* − 3	8.03*e* − 5
*f*_4_	1.08*e* − 7	1.19*e* − 6	5.61*e* − 5	1.01*e* − 6
*f*_5_	2.01*e* − 9	3.15*e* − 9	4.21*e* − 7	8.17*e* − 9
*f*_6_	3.09*e* − 10	7.14*e* − 10	7.26*e* − 9	9.50*e* − 10

**Table 2:** Comparing the maximum value of H-PSO-DE with respect to the other state-of-the-art algorithms.

Function	Maximum value of the solution			
	H-PSO-DE	H-PSO	DE	PSO-DE
*f*_1_	6.35*e* − 8	8.74*e* − 7	6.20*e* − 3	4.81*e* − 6
*f*_2_	5.94*e* − 7	6.61*e* − 7	7.24*e* − 4	1.25*e* − 6
*f*_3_	7.12*e* − 6	4.91*e* − 5	6.16*e* − 2	2.73*e* − 4
*f*_4_	4.25*e* − 7	3.46*e* − 6	4.91*e* − 3	2.51*e* − 6
*f*_5_	4.34*e* − 9	5.24*e* − 9	7.61*e* − 5	9.42*e* − 9
*f*_6_	5.32*e* − 10	1.81*e* − 9	2.81*e* − 8	1.05*e* − 9

**Table 3:** Comparing the minimum value of H-PSO-DE with respect to the other state-of-the-art algorithms.

Function	Minimum value of the solution			
	H-PSO-DE	H-PSO	DE	PSO-DE
*f*_1_	9.34*e* − 9	5.21*e* − 7	5.28*e* − 7	5.94*e* − 8
*f*_2_	1.56*e* − 7	4.26*e* − 7	3.92*e* − 7	2.63*e* − 7
*f*_3_	8.35*e* − 7	2.74*e* − 5	1.97*e* − 4	6.91*e* − 6
*f*_4_	8.61*e* − 8	7.54*e* − 7	3.71*e* − 6	3.08*e* − 7
*f*_5_	5.97*e* − 10	2.01*e* − 9	8.87*e* − 8	9.86*e* − 10
*f*_6_	1.23*e* − 10	3.51*e* − 10	6.24*e* − 10	2.37*e* − 10

## Appendix

### Benchmark Functions

Sphere:

(A.1) $$\begin{array}{c} f_{1}\left(x\right)=\overset{30}{\underset{i=1}{\sum }}x_{i}^{2},\;-50\leq x_{i}\leq 50,\\ \min\left(f_{1}\right)=f_{1}\left(0,\ldots ,0\right)=0. \end{array}$$

Rosenbrock:

(A.2) f2(x)=∑i=129(100(xi+1−xi2)2+(xi−1)2),       −30≤xi≤30,min⁡(f2)=f2(1,…,1)=0.

Rastrigin:

(A.3) f3(x)=∑i=130(xi2−10cos⁡(2πxi)+10),       −5.12≤xi≤5.12,min⁡(f3)=f3(0,…,0)=0.

Griewank:

(A.4) f4(x)=14000∑i=130xi2−Πi=130cos⁡(xii)+1,         −600≤xi≤600,min⁡(f4)=f4(0,…,0)=0.

Ackley:

(A.5) f5(x)=−20exp⁡(−0.2130∑i=130xi2) −exp⁡(130∑i=130cos⁡2πxi)        −32≤xi≤32,min⁡(f5)=f5(0,…,0)=0.

Schaffer's F6:

(A.6) f6(x)=0.5+(sinx12+x22)−0.5(1+0.001(x12+x22))2,       −100≤xi≤100,min⁡(f6)=f6(0,0)=0.

## References

[1] Zhang C, Ning J, Lu S, Ouyang D, Ding T (2009). A novel hybrid differential evolution and particle swarm optimization algorithm for unconstrained optimization. *Operations Research Letters*.

[2] Kennedy J, Eberhart R Particle swarm optimization.

[3] Zhang J, Wang J, Yue C (2012). Small population-based particle swarm optimization for short-term hydrothermal scheduling. *IEEE Transactions on Power Systems*.

[4] Bhattacharya R, Bhattacharyya TK, Garg R (2012). Position Mutated hierarchical particle swarm optimization and its application in synthesis of unequally spaced antenna arrays. *IEEE Transactions on Antennas and Propagation*.

[5] Cavuslua MA, Karakuzub C, Karakayac F (2012). Neural identification of dynamic systems on FPGA with improved PSO learning. *Applied Soft Computing*.

[6] Han M, Fan J, Wang J (2011). A dynamic feedforward neural network based on gaussian particle swarm optimization and its application for predictive control. *IEEE Transactions on Neural Networks*.

[7] Peng F, Tang K, Chen G, Yao X (2010). Population-based algorithm portfolios for numerical optimization. *IEEE Transactions on Evolutionary Computation*.

[8] Li X, Yao X (2012). Cooperatively coevolving particle swarms for large scale optimization. *IEEE Transactions on Evolutionary Computation*.

[9] Li M, Lin D, Kou J (2012). A hybrid niching PSO enhanced with recombination-replacement crowding strategy for multimodal function optimization. *Applied Soft Computing Journal*.

[10] Janson S, Middendorf M (2005). A hierarchical particle swarm optimizer and its adaptive variant. *IEEE Transactions on Systems, Man, and Cybernetics B*.

[11] Epitropakis MG, Tasoulis DK, Pavlidis NG, Plagianakos VP, Vrahatis MN (2011). Enhancing differential evolution utilizing proximity-based mutation operators. *IEEE Transactions on Evolutionary Computation*.

[12] Blum C, Puchinger J, Raidl GR, Roli A (2011). Hybrid metaheuristics in combinatorial optimization: a survey. *Applied Soft Computing Journal*.

[13] Price KV, Storn R, Lampinen J (2005). *Differential Evolution: A Practical Approach to Global Optimization*.

[14] Epitropakis MG, Plagianakos VP, Vrahatis MN (2012). Evolving cognitive and social experience in particle swarm optimization through differential evolution: a hybrid approach. *Information Sciences*.

[15] Xin B, Chen J (2011). A survey and taxonomy on hybrid algorithms based on particle swarm optimization and differential evolution. *Journal of Systems Science and Mathematical Sciences*.

[16] Liu H, Wang X, Tan G (2006). Convergence analysis of particle swarm optimization and its improved algorithm based on chaos. *Control and Decision*.

[17] Jiang S, Wang Q, Jiang J Particle swarm optimization algorithm based on velocity differential evolution.

[18] Ghosh S, Das S, Kundu D, Suresh K, Abraham A (2012). Inter-particle communication and search-dynamics of lbest particle swarm optimizers: an analysis. *Information Sciences*.

[19] Xie XF, Zhang WJ, Yang ZL A dissipative particle swarm optimization.

[20] Gao W, Liu S, Huang L (2012). Particle swarm optimization with chaotic opposition-based population initialization and stochastic search technique. *Communications in Nonlinear Science and Numerical Simulation*.

[21] Janson S, Middendorf M (2006). A hierarchical particle swarm optimizer for noisy and dynamic environments. *Genetic Programming and Evolvable Machines*.

[22] Neri F, Tirronen V (2010). Recent advances in differential evolution: a survey and experimental analysis. *Artificial Intelligence Review*.

[23] Trelea IC (2003). The particle swarm optimization algorithm: convergence analysis and parameter selection. *Information Processing Letters*.

